# Genome-Wide Characterization and Heat Responsive Expression Profiling of the HSP10 Co-Chaperonin Gene Family in *Brassica juncea*

**DOI:** 10.3390/ijms27198640

**Published:** 2026-09-27

**Authors:** Wajid Saeed, Zhangrong Chen, Samavia Mubeen, Sana Basharat, Qiqi Peng, Huiping Zhao, Muzammal Rehman, Yun Li, Muhammad Waseem, Pingwu Liu

**Affiliations:** 1 School of Breeding and Multiplication (Sanya Nanfan Research Institute of Breeding and Multiplication), College of Tropical Agriculture and Forestry, Hainan University, Sanya 572025, China; wajidsaeed@hainanu.edu.cn (W.S.); zhangrongchen@hainanu.edu.cn (Z.C.); sanabasharat143@gmail.com (S.B.); 996427@hainanu.edu.cn (H.Z.); 2 Guangxi Key Laboratory of Agro-Environment and Agric Products Safety, Key Laboratory of Plant Genetics and Breeding, College of Agriculture, Guangxi University, Nanning 530004, China; samavia.mubeen@gmail.com (S.M.); manuscript02@outlook.com (Q.P.); gxuliyun@gxu.edu.cn (Y.L.); 3 Key Laboratory of Salt-Tolerant Crops Biotechnology of Hainan Province, Sanya 572025, China; 4 International Center for Interdisciplinary Research in Sciences (ICIRS), The University of Lahore, Lahore 54000, Pakistan; muzammalr901@gmail.com

**Keywords:** *Brassica juncea*, heat shock protein 10 (HSP10), heat stress tolerance, gene family characterization, co-chaperonin

## Abstract

Heat stress disrupts protein homeostasis, yet the thermoregulatory roles of HSP10/CPN10 co-chaperonins in polyploid crops remain unexplored. This study conducted the first genome-wide characterization of the *HSP10* gene family in allotetraploid *Brassica juncea* to identify heat tolerance candidates. Genome-wide identification of *BjuHSP10* genes was performed across A and B subgenomes, followed by phylogenetic, gene structure, duplication, synteny, promoter, and structural analyses. Transcriptional responses were evaluated under moderate (28/24 °C) and severe (36/32 °C) heat stress conditions. Ten *BjuHSP10* genes were identified, encoding six mitochondrial, three chloroplast-targeted, and one cytoplasmic protein. Mitochondrial members were small (~10.7 kDa) with three-exon structures, whereas chloroplast members were larger (15.5–27.1 kDa) with complex exon–intron organization. Segmental duplication drove family expansion (19 duplicated pairs), with differential retention of *Arabidopsis* homologs. Promoter regions contained 391 putative cis-regulatory elements associated with stress, hormonal, and developmental regulation. Under heat stress, *BjuHSP10*-4 and *BjuHSP10*-5 were induced under both treatments; *BjuHSP10*-1/3/6/9 responded only to severe stress, while four genes were repressed. Structural modeling confirmed conserved β-rich GroES/CPN10-like folds. These findings identify candidate *BjuHSP10* genes, particularly *BjuHSP10*-4 and *BjuHSP10*-5, as promising targets for heat-tolerance improvement in mustard, providing a foundation for future breeding and functional studies in polyploid crops.

## 1. Introduction

High temperature stress is an increasingly important constraint on agricultural productivity because even relatively short episodes of elevated temperature can disrupt plant growth, reproduction, photosynthesis, respiration, and grain or seed development [1,2,3]. Unlike motile organisms, plants cannot avoid adverse thermal conditions; therefore, their survival depends on rapid physiological, biochemical, and transcriptional adjustments [4,5]. Heat stress destabilizes cellular membranes, alters enzyme kinetics, increases reactive oxygen species (ROS) production, impairs electron transport, and promotes the unfolding or aggregation of proteins. Because the maintenance of a correctly folded and functional proteome is essential for all cellular processes, protein quality control is a central component of plant thermotolerance [1,6,7,8].

Heat-shock proteins (HSPs), together with heat-shock transcription factors (HSFs), antioxidant enzymes, osmoprotectants, and phytohormone-mediated signaling pathways, form an integrated defense system that enables plants to sense, withstand, and recover from thermal stress [9,10]. HSPs are molecular chaperones that assist protein folding and proteostasis under normal and stress conditions [11]. They do not generally encode structural information for the final protein conformation; rather, they prevent inappropriate intermolecular interactions, promote refolding of non-native proteins, facilitate assembly of multisubunit complexes, and assist in the degradation or sequestration of irreversibly damaged proteins [12,13,14]. Plant HSPs are commonly classified according to their approximate molecular masses into HSP100, HSP90, HSP70, HSP60/chaperonins, HSP40/DnaJ proteins, and small heat-shock proteins (sHSPs) [15,16]. Although HSP10 are sometimes grouped with sHSPs on the basis of their low molecular mass, HSP10/CPN10 proteins are structurally and evolutionarily distinct from canonical sHSPs. Canonical sHSPs are defined by a conserved α-crystallin domain and generally function as ATP-independent chaperones that sequester aggregation-prone client proteins. In contrast, HSP10/CPN10 is a GroES-type co-chaperonin that functions with type I HSP60/CPN60 chaperonins [17,18,19,20]. The type I chaperonin system, comprising HSP60/CPN60 and its co-chaperonin HSP10/CPN10, is a major protein-folding machinery in mitochondria and chloroplasts. HSP60/CPN60 assembles into a double-ring cylinder, whereas HSP10/CPN10 forms a heptameric cap that binds the ATP-bound chaperonin and encloses a folding chamber for non-native substrates [20,21]. This ATP-dependent reaction cycle limits aggregation and promotes productive folding of selected imported or non-native proteins [22]. The system is particularly important in organelles of endosymbiotic origin, because numerous nuclear-encoded proteins are imported into mitochondria and chloroplasts in unfolded or incompletely folded states and must attain their native conformation after translocation [23]. In mitochondria, HSP10 cooperates with HSP60 to support protein folding in the matrix, whereas chloroplast CPN10/CPN20 co-chaperonins function with CPN60 to sustain plastid protein homeostasis [24]. Mitochondria and chloroplasts are highly sensitive to heat stress: mitochondrial perturbation affects respiration, membrane potential, ROS metabolism, and redox homeostasis, while heat disrupts photosynthetic activity and membrane-associated processes in chloroplasts [25]. Organellar protein-quality-control systems are therefore important determinants of plant thermotolerance [26,27]. Experimental evidence supports the functional importance of HSP10/CPN10 co-chaperonins in plants. A CPN10 homolog was identified in potato mitochondria through its ATP-dependent interaction with CPN60 [28], while heat-shock experiments in barley identified organellar 10 and 12 KDa proteins with homology to CPN10 [29]. In *Arabidopsis thaliana*, the mitochondrial CPN10 protein encoded by *AT1G14980* complements an *Escherichia coli* GroES mutant, and its transcript is upregulated by heat-shock treatment [30]. In pearl millet (*Pennisetum glaucum*), *PgHSP10* was cloned and characterized as a heat-responsive gene, and functional promoter analysis demonstrated that the *PgHSP10* promoter contains cis-regulatory motifs associated with abiotic-stress responsiveness and exhibits strong activity under heat stress [31]. Comparative evolutionary analysis further indicates that plant CPN10 proteins retain conserved core residues while showing diversification in sequence features, predicted localization, expression patterns, and potential functional interactions [32]. Nevertheless, HSP10/CPN10 has received substantially less genome-wide attention than other HSPs families. This under representation, coupled with the essential role of HSP10 in proteostasis, highlights the need for species-specific family-level investigations.

*Brassica juncea* (L.) is an important oilseed, condiment, vegetable, and forage crop cultivated across diverse agroecological regions. It is an allotetraploid species with an AABB genome originating from hybridization between *Brassica rapa* (AA) and *Brassica nigra* (BB), followed by chromosome doubling [33]. Its allopolyploid origin provides a valuable framework for studying gene-family evolution because duplicated homoeologous genes may be retained, lost, transcriptionally diverged, or functionally specialized following polyploidization. Genome sequencing and population genomic analyses have revealed a complex history of domestication, subgenome evolution, and structural variation in *B. juncea* [34]. Such genomic complexity is likely to affect the copy number, chromosomal distribution, sequence conservation, promoter composition, and expression patterns of stress-associated genes, including HSP10 family members. Despite the agricultural relevance of *B. juncea* and the recognized importance of chaperonin systems in organellar proteostasis, a systematic genome-wide characterization of the HSP10 gene family in this species has not been comprehensively reported. In the present study, we conducted a genome-wide identification and characterization of the HSP10 gene family in *B. juncea.* The study integrated conserved-domain validation, physicochemical characterization, subcellular-localization prediction, phylogenetic analysis, exon–intron organization, motif distribution, chromosomal mapping, gene-duplication analysis, and synteny analysis with *A. thaliana* and *O. sativa*. Promoter regions were analyzed to identify putative hormone, stress, and growth-responsive cis-regulatory elements, while secondary and tertiary structural modeling was used to examine the conservation of characteristic CPN10/GroES features. In addition, heat treatment experiments and quantitative real-time PCR were employed to investigate the transcriptional responses of *BjuHSP10* genes under moderate and severe heat stress. This integrated analysis provides a molecular and evolutionary framework for understanding HSP10 diversity in allotetraploid mustard and identifies candidate genes for future functional validation and heat-tolerance breeding.

## 2. Results

### 2.1. Identification and Physicochemical Characterization of BjuHSP10 Genes

A total of 10 HSP10 genes were identified in the *B. juncea* genome and designated as *BjuHSP10*-1 to *BjuHSP10*-10 based on their genomic positions and sequence homology. These genes were subjected to physicochemical and subcellular localization analyses to explore their structural diversity and potential functional specialization (Table 1). Among the identified BjuHSP10 members, six genes (BjuHSP10-3/4/5/6/7/9) were predicted to localize in mitochondria. These mitochondrial BjuHSP10 proteins were relatively small in length (97–98 amino acids) with molecular weights (10.66–10.81 kDa). Despite their shared localization, they exhibited heterogeneity in their isoelectric points (pI) (6.73–7.99). Notably, while all mitochondrial members were predicted to be stable proteins (instability index < 40.0), they possessed high aliphatic indices (99.29–107.42), suggesting thermal stability. Their negative GRAVY scores (−0.211 to −0.336) indicated a hydrophilic nature, typical of proteins that function in the aqueous matrix of the organelle. Three BjuHSP10 members (BjuHSP10-1/8/10) were predicted to be localized in chloroplasts. These proteins showed greater variation in size (144–256 amino acids) with molecular weights (15.54–27.09 kDa). The theoretical pI values ranged (5.34–8.87), with BjuHSP10-1 and BjuHSP10-8 exhibiting alkaline properties, whereas BjuHSP10-10 showed an acidic nature. Their instability indices ranged (28.03–39.28) and aliphatic indices ranged (89.31–98.24), indicating relatively stable proteins with potential thermal stability. The GRAVY values remained negative (−0.061 to −0.100), further confirming their hydrophilic characteristics.

One member, BjuHSP10-2, was predicted to localize in the cytoplasm and displayed distinct structural characteristics compared with other family members. It encoded a large protein (957 amino acids) with a molecular weight (108.78 kDa) and showed the highest theoretical pI value (9.35). The protein exhibited an instability index (38.78), an aliphatic index (94.01), and a GRAVY value (−0.304), indicating a stable and hydrophilic protein. Overall, chloroplast-targeted members were generally larger and less hydrophilic than the mitochondrial proteins, whereas mitochondrial *BjuHSP10* members were small, compact, and stable, with high aliphatic indices suggesting good thermal stability. In contrast, the predicted cytoplasmic member was exceptionally large and more hydrophilic but remained stable, as indicated by its instability index (38.78), distinguishing it from the typical small HSP10 proteins localized in mitochondria and chloroplasts.

### 2.2. Gene Structure, Conserved Motifs, and Domain Organization of BjuHSP10 Family Genes

To investigate the structural diversity and evolutionary conservation of the *B. juncea* HSP10 family, the exon–intron organization, conserved motifs, and conserved protein domains of the identified BjuHSP10 members were analyzed (Figure 1). The unrooted phylogenetic clustering grouped the BjuHSP10 proteins into five distinct groups, which were largely consistent with their gene structures and conserved motif compositions. Conserved domain analysis revealed that all BjuHSP10 proteins contained characteristic HSP10/chaperonin-related domains, but their domain compositions differed among proteins. The major conserved domains identified included CPN10, GroES, and P-loop NTPase superfamily-related domains. The mitochondrial-associated members (BjuHSP10-3/4/5/6/7/9) and one chloroplast associated member (BjuHSP10-5) mainly contained conserved CPN10/cpn10 domain, reflecting their conserved chaperonin function. The chloroplast-localized members (BjuHSP10-1/8) possessed dual GroES/groES domain. The dual naming of these domains reflects the simultaneous detection of the same conserved region by both a specific/curated model (uppercase initial) and a broader superfamily model (lowercase initial) in the NCBI Conserved Domain Database (CDD), rather than indicating two distinct functional domains. In contrast, BjuHSP10-2 showed the most complex domain organization, containing two separated conserved regions (CPN10 and P-loop NTPase superfamily) distributed across its extended protein sequence.

Analysis of exon–intron organization revealed marked structural variation among the *BjuHSP10* family members. The mitochondrial-localized members (*BjuHSP10*-3/4/5/6/7/9) exhibited relatively compact genomic architectures, each comprising three exons and two introns. The chloroplast-targeted genes *BjuHSP10*-1 and *BjuHSP10*-8 shared a highly conserved organization, harboring five exons and five introns with similar distribution patterns. In contrast, *BjuHSP10*-10 displayed a distinct configuration, containing five exons and four introns. The cytoplasmic member *BjuHSP10*-2 emerged as the most structurally divergent members, possessing a substantially large genomic region with ten exons and nine introns, a feature that correlates with its exceptionally large protein size. Conserved motif analysis identified eight motifs (Motif 1–8) among BjuHSP10 proteins. Motif 2 was universally conserved in all ten members, while Motif 1 and Motif 7 were present in nine members, indicating their essential roles in HSP10 structural conservation. Mitochondrial-localized BjuHSP10 members (BjuHSP10-3/4/5/6/7/9) shared a highly conserved core motif arrangement (Motif 2-7-1). Among these, BjuHSP10-3/4/6/7/9 additionally contained Motif 3, whereas BjuHSP10-5 lacked this motif, indicating minor structural variation within the mitochondrial group. Chloroplast members BjuHSP10-1 and BjuHSP10-8 exhibited an identical motif pattern (Motif 7-6-2-4-5-1-8), whereas BjuHSP10-10 showed a highly reduced motif composition containing only universal Motif 2. The cytoplasmic isoform BjuHSP10-2 displayed a unique motif arrangement (Motif 5-2-7-1-3) that differed from other family members, further underscoring its evolutionary divergence.

### 2.3. Phylogenetic Analysis of HSP10 Proteins with Other Proteins

To investigate the evolutionary relationships of *B. juncea* HSP10-related proteins with homologues from representative dicot and monocot species, a maximum-likelihood phylogenetic tree was reconstructed using a filtered alignment of 22 protein sequences: 10 BjuHSP10, five *A. thaliana* HSP10, and seven *O. sativa* HSP10-related proteins (Figure 2). The phylogenetic analysis classified the HSP10 proteins into five major groups (I–V), revealing both conserved evolutionary lineages and *B. juncea*-specific diversification. Group I (pink) comprised OsHSP10-7, AtHSP10-3/4 and BjuHSP10-10, representing a mixed-species clade. Group II (blue) contained OsHSP10-1/4/3/6, BjuHSP10-1/8, and AtHSP10-5, indicating strong evolutionary conservation among homologous HSP10 proteins. Group III (yellow) included OsHSP10-5/2, AtHSP10-1/2, and BjuHSP10-5, representing another conserved lineage. Group IV (red) consisted solely of BjuHSP10-2, which formed an independent branch, indicating an early divergence and distinct evolutionary trajectory. Group V (purple) was composed exclusively of *B. juncea* HSP10 proteins (BjuHSP10-3/4/6/7/9), forming a species-specific cluster that likely resulted from lineage-specific duplication and diversification events during *B. juncea* evolution. The close association of BjuHSP10 proteins with AtHSP10 proteins in Groups I-III suggests their origin from common ancestral genes and potential functional conservation.

### 2.4. Chromosomal Distribution of BjuHSP10 Genes

Analysis of chromosomal distribution revealed that the ten *BjuHSP10* genes were unevenly localized across the *B. juncea* genome, with six genes on the A-subgenome (chromosomes A02, A06, A08, and A09) and four on the B-subgenome (B03, B05, and B06) (Figure 3). Specifically, *BjuHSP10*-1 resided on A02; *BjuHSP10*-2 on A06; *BjuHSP10*-3 on A08; *BjuHSP10*-4 and *BjuHSP10*-5 on A09; *BjuHSP10*-6 and *BjuHSP10*-7 on B03; *BjuHSP10*-8 on B05; and *BjuHSP10*-9 and *BjuHSP10*-10 on B06. The presence of duplicated homologous pairs of chromosomes A09, B03 and B06 suggests that genome duplication events and subsequent gene retention have contributed to the expansion of the *BjuHSP10* family. Collectively, this dispersed distribution pattern reflects a complex evolutionary history involving duplication and lineage-specific diversification within the *B. juncea* genome.

### 2.5. Synteny Analysis: Intra-Species Duplication and Inter-Species Orthology

In the present study, a total of 19 segmental duplications and one tandem duplication were detected among the *BjuHSP10* genes (Figure 4a, Table S2). These duplicated gene pairs were distributed across chromosomes A02, A06, A08, A09, B03, B05, and B06, involving both the A and B subgenomes. The identified segmental duplications included *BjuHSP10*-1/2/3/4/6/7/8/9, which showed collinear relationships with homologous genomic regions. The predominance of segmental over tandem duplications, indicates that polyploidization-driven genome duplication was the primary mechanism responsible for the expansion and diversification of *HSP10* genes in *B. juncea*.

Inter-species synteny analysis was performed between *B. juncea* and *A. thaliana,* and between *B. juncea* and *O. sativa* (Figure 4b, Table S3). No syntenic relationships were detected with *O. sativa*, which is consistent with the substantial evolutionary divergence between the *Brassicaceae* and *Poaceae* lineages. Synteny analysis between *B. juncea* and *A. thaliana* identified 11 syntenic gene pairs involving four *A. thaliana* loci and seven *B. juncea* chromosomes, indicating strong evolutionary conservation of *HSP10* genes. Among these, seven gene pairs represented single-copy orthologous relationships, whereas several loci showed evidence of duplication and differential retention. The *Arabidopsis* locus AT1G14980.1 exhibited the highest conservation, forming syntenic relationships with six *B. juncea* genes distributed across chromosomes A06, A08, A09, B03, and B06, suggesting extensive retention of this ancestral HSP10 locus after genome evolution. In contrast, *BjuHSP10*-10 displayed co-orthologous relationships with AT2G44650.1 and AT3G60210.1, indicating localized gene duplication. Additionally, AT5G20720.1 showed conserved multi-copy retention in both *BjuHSP10*-1 and *BjuHSP10*-8, suggesting preservation of ancestral duplicated copies in the A and B subgenomes. The distribution of syntenic blocks was uneven among chromosomes, with B06 showing the highest complexity, followed by A09 and B03, which each contained two syntenic loci. These results indicate that duplication events and differential gene retention during polyploid evolution have contributed to the expansion and diversification of the BjuHSP10 family.

### 2.6. Cis-Acting Regulatory Element Prediction for the BjuHSP10 Genes

To explore the potential transcriptional regulation of the *BjuHSP10* family, the 2000 bp upstream promoter regions of all genes were analyzed using Plant Cis-Acting Regulatory Element (PlantCARE) database. A total of 391 cis-regulatory elements were identified and classified into stress-responsive (136), hormone-responsive (133), and growth- and development-related (122) categories (Figure 5). Among the 10 stress-responsive element types, myelocytomatosis (MYC) motifs (stress-responsive) were detected in all *BjuHSP10* promoters, with multiple copies in most genes, whereas *BjuHSP10*-10 contained only one copy. Myeloblastosis (MYB)-binding sites (stress-responsive) were the second most abundant elements, with the highest numbers in *BjuHSP10*-5 (six copies) and *BjuHSP10*-9 (four copies). Anaerobic Response Element (ARE) elements (anaerobic response) being also widely distributed, occurring at one to four copies across multiple promoters.

Among the 10 hormone-responsive element types, Abscisic Acid Response Elements (ABREs) were particularly enriched in *BjuHSP10*-2 (14 copies) and *BjuHSP10*-7 (six copies). The CGTCA and TGACG motifs (methyl jasmonate response) and as-1 elements (auxin response) were also abundant in *BjuHSP10*-2 (six copies each) and *BjuHSP10*-7 (five copies each), while Ethylene Response Elements (EREs) were most abundant in *BjuHSP10*-8 (five copies). Among the 15 growth- and development-related element types, the G-box (light response) was enriched in *BjuHSP10*-3 (seven copies) and *BjuHSP10*-7 (four copies), whereas Box 4 (light response) was most abundant in *BjuHSP10*-4, *BjuHSP10*-8, and *BjuHSP10*-9 (four copies each). The TCT motif (light response) was enriched in *BjuHSP10*-5 and *BjuHSP10*-7 (four copies each). Overall, the diverse distribution of stress-, hormone-, and light-responsive cis-elements suggests that *BjuHSP10* genes may participate in complex regulatory networks integrating environmental and hormonal signals.

### 2.7. Predicted Structures of BjuHSP10 Proteins

To gain structural insight into the BjuHSP10 protein family, homology-based tertiary structure modeling was performed using the SWISS-MODEL platform. Nine of the ten BjuHSP10 proteins yielded high-confidence models, with GMQE (Global Model Quality Estimation) scores (0.71–0.91) and sequence identity exceeding (90%) relative to the selected *A. thaliana* templates in most cases (Figure 6, Table S4). Based on the best-fit templates, the BjuHSP10 proteins were structurally classified into two distinct organellar groups: a mitochondrial-type group (BjuHSP10-3/4/5/6/7/9), modeled on the 10 kDa mitochondrial chaperonin (P34893 or Q8LDC9), and a chloroplastic-type group (BjuHSP10-1/8/10), modeled on the 20 kDa chloroplastic chaperonins (O65282 or O80504). Notably, the predicted secondary structures were highly conserved across all nine members, characterized by a marked predominance of β-strands (32.4–38.1%) and random coils (58.8–64.1%), with only negligible α-helical content (<6.2%). This structural composition is entirely consistent with the canonical β-barrel “jelly-roll” fold that is archetypal of the CPN10 (GroES) family, underscoring the high structural integrity of these predicted proteins. In contrast, BjuHSP10-2 (957 aa) deviated substantially, modeling against a helicase C-terminal domain-containing protein (M4EQE8) with reduced coverage (74.2%) and a comparatively low GMQE of 0.48, suggesting that this locus likely represents a distinct protein class within the BjuHSP10 protein inventory.

Homology modeling by SWISS-MODEL produced confident models (GMQE ≥ 0.9) for all ten BjuHSP10 proteins. Notably, five of them, BjuHSP10-3/4/6/7/9, adopted essentially identical folds, with pairwise Cα RMSD ≤ 0.01 Å, consistent with their high sequence identity (93.9–98.0%; Table S4). The few substitutions among these paralogs (e.g., Val → Ile, Val → Leu, Gln → Lys) are conservative and do not perturb the conserved sHSP/α-crystallin fold, indicating strong purifying selection on the chaperone domain since their recent duplication. By contrast, BjuHSP10-5 deviated from this group (Cα RMSD 9.6 Å), BjuHSP10-10 formed a distinct structure, BjuHSP10-1 and 8 each carry N-terminal extensions that appear as extended loops, and BjuHSP10-2 is a multi-domain protein whose template-based model covered 710 of its 957 residues.

### 2.8. Expression Profiling of BjuHSP10 Genes Under Heat Stress by qPCR

To elucidate the transcriptional response of the *BjuHSP10* gene family to thermal stress, qRT-PCR was performed on leaf tissue subjected to moderate temperature stress (MTS) and high temperature stress (HTS), with expression levels normalized to CK conditions. The analysis revealed striking and gene-specific expression patterns among the ten *BjuHSP10* genes (Figure 7). A subset of genes, *BjuHSP10*-4 and *BjuHSP10*-5, displayed pronounced and constitutive upregulation under both MTS and HTS, indicating a broad-spectrum response to temperature elevation. In contrast, *BjuHSP10*-1/3/6/9 were specifically and markedly induced under HTS, while their transcript levels under MTS were either comparable to or lower than the control, suggesting a selective activation under severe heat conditions. Conversely, a distinct set of four genes, *BjuHSP10*-2/7/8/10, exhibited significant repression across both stress treatments, with particularly notable reductions in transcript abundance observed for *BjuHSP10*-7 and *BjuHSP10*-10. Collectively, these findings demonstrate that the *BjuHSP10* family in *B. juncea* leaves comprises functionally diverse genes, with specific genes acting as potential positive regulators under heat stress while others are suppressed, highlighting the complexity and specificity of the plant’s transcriptional adaptation to varying temperature regimes.

## 3. Discussion

The present study provides the first genome-wide analysis of the *HSP10*/co-chaperonin gene family in *B. juncea*, extending knowledge of this small but functionally important chaperonin component from model plants to an allotetraploid oilseed crop. Rather than representing a uniform set of heat-shock proteins, the ten BjuHSP10 candidates appear to comprise structurally and evolutionarily distinct proteins that may support organelle-specific proteostasis. This interpretation is consistent with the central role of Group I chaperonin systems in mitochondria and chloroplasts, where HSP10/CPN10-like co-chaperonins act as detachable caps for HSP60/CPN60 folding chambers and help protect non-native polypeptides during folding [32,35]. The marked contrast between the small, three-exon, mitochondrial-predicted BjuHSP10 proteins and the larger, structurally more complex chloroplast-predicted members is therefore biologically meaningful, because chloroplast chaperonin systems have evolved greater subunit diversity than their mitochondrial counterparts to accommodate the folding demands of photosynthesis and plastid development [32]. The mitochondrial-localized BjuHSP10-3/4/5/6/7/9 proteins were highly similar in size to the conventional approximately 10 kDa CPN10 proteins described in bacteria and mitochondria [36]. Their compact architecture supports the hypothesis that these proteins are canonical co-chaperonins rather than merely HSP10-domain-containing proteins. In *A. thaliana*, mitochondrial HSP10 proteins participate in the organellar chaperonin system, whereas chloroplasts contain both conventional CPN10 proteins and CPN20, a distinctive co-chaperonin with two tandem CPN10-like domains [24,32,35]. The larger size and more complex exon–intron organization of BjuHSP10-1/8/10 are therefore compatible with chloroplast-specific diversification, potentially reflecting acquisition of transit peptides, duplicated CPN10-like domains, or regulatory sequences associated with plastid functions. Because chloroplast chaperonins are involved in the folding of Rubisco large subunits and other photosynthetic proteins, structural diversification among chloroplast-targeted *BjuHSP10* genes may have consequences for photosynthetic stability under heat stress [24,37]. In contrast, BjuHSP10-2 was predicted to encode a 957-amino-acid, approximately 109 kDa cytoplasmic protein containing both CPN10-related and P-loop NTPase-associated domains. Its extreme length, domain composition, and divergent phylogenetic placement are more consistent with either a multi-domain protein that has retained a CPN10-like region or a gene-model annotation error. The apparent P-loop NTPase domain raises the possibility of ATP-dependent remodeling or trafficking activity, but this possibility remains hypothetical because canonical HSP10 co-chaperonins do not function as ATPases; ATP binding and hydrolysis occur primarily through their HSP60/CPN60 partners [38]. Targeted transcript validation, full-length cDNA sequencing, and localization assays should therefore be prioritized before assigning BjuHSP10-2 protein a role in co-chaperonin-mediated heat tolerance.

Phylogenetic and synteny analyses indicate that the BjuHSP10 family has been shaped by both conservation and duplication-driven diversification. This pattern of conserved and lineage-specific clades is consistent with previous phylogenetic analyses of *HSP60/10* gene families in other plant species. For instance, ref. [39] identified 36 *Hsp60/10* genes in *Sorghum bicolor* that were subdivided into two groups corresponding to HSP60 and HSP10 co-chaperonins, with segmental duplication being the predominant mechanism of family expansion. Similarly, in *P. glaucum*, phylogenetic analysis revealed that *PgHSP10* is evolutionarily close to the maize HSP10 homolog, suggesting conserved orthologous relationships among cereal HSP10 genes [31]. The clustering of several *BjuHSP10* proteins with *A. thaliana* HSP10/CPN10 homologues supports retention of ancestral co-chaperonin functions within *Brassicaceae*. However, the *B. juncea*-enriched Group V clade, containing BjuHSP10-3/4/6/7/9, suggests that not all duplicated copies have remained functionally equivalent. This pattern is consistent with the evolutionary history of *B. juncea* as an allotetraploid species carrying A and B subgenomes, in which duplicated loci may be retained, lost, transcriptionally partitioned, or functionally specialized after polyploidization. Similar transcriptional subfunctionalization of duplicated genes has been reported in *B. juncea*, demonstrating that polyploidy can preserve multiple related copies while allowing condition- and tissue-specific expression divergence [40]. Consistent with this evolutionary framework, comparative synteny analysis further revealed that the *A. thaliana* mitochondrial co-chaperonin locus At1g14980 was associated with six *BjuHSP10* homologues distributed on chromosomes A06, A08, A09, B03, and B06, demonstrating retention of multiple homologous loci in the *B. juncea* genome. The non-uniform distribution of these syntenic relationships, including the large number and/or complexity of syntenic connections on chromosome B06, is compatible with differential retention and post-polyploid genome fractionation [41]. The syntenic complexity on chromosome B06 may reflect the specific evolutionary trajectory of the B subgenome in *B. juncea*, which has been shaped by cytonuclear interactions and subgenome dominance following allopolyploidization [42]. However, establishing subgenome dominance will require genome-wide evaluation of homeolog retention and expression bias. Similar contributions of whole-genome/segmental duplication to gene-family expansion, followed by differential retention and functional divergence of duplicated genes, have been widely documented in *Brassica* genomes [43,44] and in other plant taxa. In *S. bicolor*, among 12 paralogous *HSP60/10* gene pairs, only one was tandem and the remaining 11 were segmental, indicating that segmental duplication was the primary driver of family expansion [39]. Analysis of duplication patterns identified 19 segmentally duplicated and one tandemly duplicated *BjuHSP10* gene pairs, indicating that large-scale duplication events were the predominant source of *BjuHSP10* copy-number expansion, whereas local tandem duplication made only a limited contribution. This pattern is consistent with the complex polyploid history of *B. juncea*, an allotetraploid species (AABB, 2*n* = 36) formed through hybridization between the ancestral lineages of *B. rapa* (AA) and *B. nigra* (BB), followed by chromosome doubling [45].

The secondary-structure and motif analyses collectively indicate that the conventional BjuHSP10 proteins retain the characteristic β-rich structural framework of the CPN10/GroES co-chaperonin family. This structural conservation is a hallmark of the HSP10 family across kingdoms. In *P. glaucum*, the predicted 3D model of PgHSP10 confirmed a conserved eight-stranded β-barrel with the active site between the β-barrel strands and a conserved VLLPEYGG domain sandwiched between two β-sheets [31]. Nine BjuHSP10 protein members were predicted to contain predominantly β-strands (32.4–38.1%) and coil regions (58.8–64.1%), accompanied by only a minor proportion of α-helices (<6.2%). This composition agrees with the established CPN10/GroES architecture, in which each subunit adopts an irregular β-barrel, often described as a jelly-roll-like β-rich fold [20]. The predominance of β-strands is structurally important because intermolecular β-strand pairing contributes substantially to the assembly and stability of the characteristic heptameric co-chaperonin ring [20,46]. The high GMQE scores (0.71–0.91) and sequence identity exceeding 90% relative to *A. thaliana* templates further support the reliability of the overall β-rich structural models and indicate that the selected templates are appropriate for modeling the conventional BjuHSP10 proteins. Motif analysis complements these structural observations. Motif 2 was conserved across all ten *BjuHSP10*-related sequences, whereas Motifs 1 and 7 were retained in nine members, demonstrating that a substantial proportion of the family shares conserved sequence blocks despite variation in length, domain organization, and phylogenetic placement. The extensive conservation of these motifs suggests that they form part of the structurally constrained sequence framework required to maintain the CPN10-like fold. Such conserved regions may support β-strand formation, stabilization of inter-subunit contacts within the co-chaperonin oligomer, or maintenance of local loop conformations required for productive association with CPN60/HSP60 [47].

Promoter analysis identified 391 putative cis-regulatory elements within the *BjuHSP10* upstream regions, including 136 stress-responsive, 133 hormone-responsive, and 122 growth and development-associated elements, indicating that the *BjuHSP10* family is embedded within a broad transcriptional regulatory network. This finding is consistent with promoter analyses of HSP gene families in other plant species. In *Triticum aestivum*, a genome-wide analysis of the HSP family identified 212 unique cis-acting regulatory elements across *TaHSP* subfamilies, with stress-responsive and transcription factor binding sites being the most abundant; these promoters contained binding motifs for MYB, MYC, and bZIP transcription factors, suggesting that *TaHSPs* play crucial roles in stress responses regulated by multiple TFs [48]. The universal occurrence of MYC motifs, together with the frequent presence of MYB-binding sites and anaerobic-response elements (AREs), suggests potential responsiveness to dehydration, oxidative, and hypoxia-associated signaling pathways, because MYC/MYB transcriptional regulators participate widely in plant abiotic-stress transcriptional responses [49,50]. The presence of MYB and MYC binding sites in *HSP10* promoters is particularly significant because these transcription factors are known to integrate multiple stress signaling pathways. For example, MYB4 and MYB29 are activated in *A. thaliana* upon combined high light and heat stress, indicating their involvement in multi-stress signaling [51]. The high ABRE abundance in *BjuHSP10*-2 (14 copies) and *BjuHSP10*-7 (six copies) further suggests that these genes may be particularly sensitive to ABA-dependent regulation. This finding is especially relevant given the well-documented crosstalk between ABA signaling and heat stress responses. In wheat, the ABA signaling pathway regulates the Hsf–Hsp module through ABF transcription factors that bind ABRE elements within HSF promoters to induce their expression [52]. Furthermore, a recent study in rice revealed that the plastid chaperonin complex *OsCpn10a*–*OsCPN20*–*OsHsp60*-3B interacts with the ABA receptor *OsPYL10*, negatively regulating ABA signaling and enhancing seed storability [52]. Under adverse conditions, ABA accumulation activates bZIP-type transcription factors that recognize ABRE-containing promoters, potentially linking water-deficit, salinity, oxidative stress, and heat-associated signals to induction of protective chaperone systems [53]. Heat-shock responses are principally governed by HSFs, but HSF activity and Hsp expression are integrated with ABA, jasmonate, salicylic acid, ethylene, and redox signaling [54]. Thus, the promoter architecture of *BjuHSP10* genes is consistent with a model in which environmental stress and phytohormone signaling converge to adjust co-chaperonin abundance and thereby preserve protein-folding capacity during cellular stress. Similarly, the occurrence of MeJA-responsive CGTCA/TGACG motifs and auxin-responsive as-1 elements in several *BjuHSP10* promoters further indicates potential coordination of HSP10 transcription with jasmonate-mediated defense and developmental signaling. Jasmonate and auxin pathways can modulate heat-response networks through crosstalk with transcription factors and HSP-associated proteostasis pathways, allowing plants to balance growth, defense, and thermotolerance under changing environmental conditions [55]. The distribution of light-responsive cis-regulatory elements across the *BjuHSP10* promoters indicates that light-associated transcriptional control is not restricted to the predicted chloroplast members. The chloroplast-localized *BjuHSP10*-8 contained Box 4 as its main light-responsive element, while the mitochondrial members collectively harbored G-box, Box 4, and TCT motifs. In chloroplast-localized *BjuHSP10*-8, light-responsive regulation could coordinate co-chaperonin availability with plastid development and photosynthetic protein-folding demand. This interpretation is consistent with the essential role of the chloroplast CPN60–CPN10/CPN20 chaperonin system in Rubisco large-subunit folding and Rubisco biogenesis [56]. Conversely, the enrichment of light-responsive elements in several predicted mitochondrial *BjuHSP10* promoters may reflect broader light-dependent coordination of organellar proteostasis, because light strongly alters cellular energy status, redox balance, metabolism, and the production of proteins requiring mitochondrial folding and quality-control systems [57]. Thus, the promoter architecture supports differential, but potentially overlapping, light-responsive regulation among *BjuHSP10* family members and may facilitate adjustment of chloroplast and mitochondrial protein-folding capacity during changing light conditions.

The qRT-PCR analysis revealed striking and gene-specific expression patterns among the ten *BjuHSP10* genes under MTS and HTS, providing functional evidence for the diversification of this gene family. The constitutive upregulation of *BjuHSP10*-4 and *BjuHSP10*-5 under both stress regimes suggests that these genes function as broad-spectrum heat-responsive chaperonins, potentially playing a primary role in maintaining protein homeostasis across a range of thermal conditions. In contrast, the selective induction of *BjuHSP10*-1/3/6/9 exclusively under severe heat stress implies a threshold-dependent activation mechanism, possibly involving HSFs that become fully activated only under extreme temperatures. The repression of *BjuHSP10*-2/7/8/10 under both stress treatments is unexpected and may reflect several non-mutually exclusive possibilities. First, these genes might be subjected to negative regulation by heat-induced transcriptional repressors, a phenomenon observed for certain *HSP* genes in other species [2,58]. Second, their downregulation might represent a resource-allocation strategy, whereby the plant prioritizes the expression of more effective chaperone members under heat stress conditions. However, the retention of these genes in the *B. juncea* genome despite their repression under heat stress suggests that they are not functionally redundant. Instead, their retention may reflect subfunctionalization or neofunctionalization, whereby duplicated copies have partitioned ancestral functions or acquired novel roles in other tissues, developmental stages, or abiotic stress responses not examined in this study. This is consistent with the known fate of duplicated genes in polyploids, where relaxed selection, dosage balance, and regulatory divergence collectively determine which copies are retained or lost [59,60]. It is also possible that these genes are in the process of pseudogenization but retain intact open reading frames due to insufficient evolutionary time since polyploidization [61]. Third, the repression might be tissue-specific, with these genes potentially showing different expression patterns in other organs such as roots, flowers, or developing seeds. Fourth, the suppressed genes might have functions that are antagonistic to thermotolerance or are not required under heat stress, consistent with the concept of functional diversification among duplicated gene copies. It is noteworthy that *BjuHSP10*-2, which contains the putative P-loop NTPase domain, was repressed under heat stress. This raises the possibility that this atypical member may function in developmental or housekeeping roles rather than in the heat-stress response, a hypothesis that warrants further investigation.

The differential expression patterns observed here are consistent with findings in other plant species. In *A. thaliana*, the mitochondrial CPN10 (AT1G14980) is markedly induced by heat shock treatment [62], analogous to the upregulation of its *B. juncea* orthologs. In *Sorghum bicolor*, specific HSP10 genes have been implicated in cold stress tolerance, indicating that chaperonin family members can acquire specialized functions in response to different abiotic stresses [39]. In *Camellia sinensis*, CsHSP60s physically interact with CsCPN10s in response to combined heat and drought stress, suggesting that HSP10 co-chaperonins function cooperatively with HSP60 partners in stress adaptation [63]. Furthermore, transgenic overexpression of a CPN10-like protein (SbCpn10L) from *Salicornia brachiata* in tobacco enhanced heat stress tolerance, associated with reduced electrolyte leakage and lower accumulation of malondialdehyde, hydrogen peroxide, and reactive oxygen species [64]. These findings collectively support the functional significance of HSP10 proteins in plant stress responses and validate the candidate genes identified in our study for future functional characterization.

## 4. Materials and Methods

### 4.1. Genome-Wide Identification and Characterization of BjuHSP10 Genes

Publicly available genome assemblies, protein sequences, and gene annotation files for *B. juncea*, *A. thaliana*, and *O. sativa* were downloaded from Ensembl Plants release 63 (https://jun2026-plants.ensembl.org/index.html, accessed 20 August 2026). The *B. juncea* reference genome ASM1870372v1, derived from the Sichuan Huangzi cultivar and provided by Hunan Agricultural University (HUNAU), was used for genome-wide identification and characterization of *HSP10* genes. For *A. thaliana*, the TAIR10 genome assembly and Araport11 gene annotation were used. For *O. sativa*, the IRGSP-1.0 genome assembly and the corresponding Ensembl Plants release 62 annotation were used. The Hidden Markov Model profile for the CPN10/GroES domain (PF00166) was obtained from the Pfam database, release 38.1 (https://ftp.ebi.ac.uk/pub/databases/Pfam/releases/, accessed 20 August 2026). Putative HSP10-encoding sequences were initially identified using the Simple HMM-Search module implemented in TBtools-II (v2.485) under default parameters. To verify the presence of the complete GroES domain, all candidate protein sequences were submitted to the NCBI Conserved Domain Database (CDD; https://www.ncbi.nlm.nih.gov/cdd/ accessed on 20 August 2026). Only those sequences harboring the full-length, characteristic chaperonin domain and falling within the expected size range for plant HSP10 proteins were retained; redundant transcripts, truncated members, and sequences with abnormal lengths were excluded. The retained candidates were further validated using BLASTP searches against the NCBI non-redundant (nr) protein database (https://blast.ncbi.nlm.nih.gov/Blast.cgi, accessed on 20 August 2026), keeping only those with top hits annotated as CPN10/GroES or related chaperone proteins. Batch computation of physicochemical parameters including amino acid length, molecular weight, theoretical isoelectric point (pI), instability index, aliphatic index, and grand average of hydropathicity (GRAVY) was performed using the Protein Parameter Calculator module in TBtools. Subcellular localization was predicted using the WoLF PSORT online server (https://wolfpsort.hgc.jp/, accessed on 20 August 2026 (accessed on)).

### 4.2. Gene Structure and Conserved Motif Analysis

The conserved protein motifs within the BjuHSP10 family were predicted using the MEME suite integrated into TBtools-II (v2.485), with the maximum number of motifs set to 20 and all remaining parameters at their default values. The exon–intron organization of each *BjuHSP10* gene was determined and graphically visualized using the Gene Structure Display Server (GSDS) (v2.0) and TBtools.

### 4.3. Phylogenetic Analysis

A total of 22 HSP10-related protein sequences, including 10 BjuHSP10 proteins from *B. juncea*, five HSP10/CPN10 proteins from *A. thaliana*, and seven proteins from *Oryza sativa*, were used for phylogenetic analysis. Protein sequences were initially aligned using ClustalW with default parameters in MEGA version 12. The initial full-length alignment comprised 962 amino-acid positions. Because HSP10-related proteins showed substantial variation in length and domain architecture, particularly the 957-amino-acid BjuHSP10-2 fusion protein containing an N-terminal P-loop NTPase region and a C-terminal CPN10/GroES domain, the alignment was filtered before tree reconstruction. Alignment columns containing gaps in more than 30% of sequences were removed; thus, only positions with residues in at least 70% of the 22 sequences were retained. This procedure yielded a final alignment of 96 amino-acid positions, including 55 fully occupied positions.

The best-fitting amino-acid substitution model was selected in MEGA 12 by comparing 64 candidate models using the Bayesian Information Criterion (BIC) and corrected Akaike Information Criterion (AICc). The LG + G + I model was selected as the optimal model (BIC = 3354.10; AICc = 3118.01; lnL = −1514.97), with an estimated gamma-shape parameter of 4.19 and an invariant-site proportion of 0.052. A maximum-likelihood phylogenetic tree was then reconstructed under the LG + G + I model. Branch support was assessed using 1000 bootstrap replicates. The resulting tree was visualized in a rectangular layout, and bootstrap values were displayed at all internal nodes.

### 4.4. Chromosomal Distribution and Synteny Analysis

Chromosomal positions of the identified *BjuHSP10* genes were extracted from the *B. juncea* genome annotation files. A chromosomal distribution map was generated using TBtools. To investigate duplication events, intra-species syntenic blocks within the *B. juncea* genome were examined using MCScanX (integrated into TBtools) based on whole-genome protein similarity searches. For inter-species collinearity analysis, the *B. juncea* HSP10 loci were compared against the genomes of *A. thaliana* and *O. sativa*. The resulting syntenic relationships were visualized as circular plots using the Advanced Circos module of TBtools.

### 4.5. Promoter Cis-Regulatory Element Analysis

The 2000 bp genomic sequences immediately upstream of the translation start site for each of the *BjuHSP10* genes were extracted from the reference genome. Putative cis-acting regulatory elements within these promoter regions were predicted using the PlantCARE database. Identified elements were manually categorized into three broad functional groups: phytohormone-responsive elements, abiotic/biotic stress-responsive elements, and elements associated with plant growth and development.

### 4.6. Protein Structure Prediction

For secondary structure prediction, the aligned sequences were submitted to the SOPMA (Self-Optimized Prediction Method with Alignment) tool with a window width of 17 residues and a similarity threshold of 8. For tertiary structure modeling, three-dimensional homology models were generated using the SWISS-MODEL server (https://swissmodel.expasy.org/), an automated web-based platform for comparative protein structure modeling. Suitable templates were selected from the Protein Data Bank (PDB) and the AlphaFold Protein Structure Database based on the highest sequence identity, optimal query coverage, and GMQE (Global Model Quality Estimation) scores. Final models were validated using QMEAN scores, and the best-fitting models were retained. Structural comparison among *BjuHSP10* members was performed by structural superposition and RMSD calculation using PyMOL v2.5, and by comparison of secondary structure composition. All structural figures were generated using PyMOL v2.5.

### 4.7. Plant Materials and Heat Stress Treatments

Seeds of *B. juncea* cultivar ‘Shanhuang’ were soaked in water at 4 °C for 48 h to break dormancy and subsequently sown in pots filled with a vermiculite-nutrient soil mixture (1:1, *v*/*v*). Plants were grown under controlled greenhouse conditions with a 16 h light (25 °C)/8 h dark (18 °C) photoperiod, a light intensity of 200 µmol m^−2^ s^−1^, and relative humidity of 65 ± 5%, and were irrigated every two days until the three-week growth stage. At this stage, uniform plants were transferred to growth chambers and subjected to three temperature regimes for seven days: a control condition (22 °C day/18 °C night), MTS (28 °C day/24 °C night), and HTS (36 °C day/32 °C night). For each treatment, three independent biological replicates were collected, with each replicate consisting of pooled fully expanded leaf samples from four randomly selected plants. All samples were immediately flash-frozen in liquid nitrogen and stored at −80 °C until further use.

### 4.8. Quantitative Real-Time PCR (qRT-PCR) Analysis

Total RNA was extracted from frozen leaf tissues using TRIzol reagent and treated with DNase I to eliminate residual genomic DNA. RNA quality was evaluated via 1.2% agarose gel electrophoresis, and concentration was measured (A260/A280 ratios) using a NanoDrop ND-1000 spectrophotometer (Thermo Fisher Scientific, Waltham, MA, USA). First-strand cDNA was synthesized using a commercial reverse transcription kit (TransGen Biotech, Beijing, China) following the manufacturer’s instructions. Gene-specific primers for each *BjuHSP10* gene were designed using Primer3 software (Table S1). The *B. juncea* β-Actin gene was used as the internal reference for qRT-PCR normalization, as its stable expression under temperature stress has been validated in previous studies [65,66]. qPCR reactions were performed on an Applied Biosystems StepOne real-time PCR system (Thermo Fisher Scientific, Waltham, MA, USA) using AceQ qPCR SYBR Green Master Mix (Vazyme, Nanjing, China) in a total volume of 20 µL, containing 10 µL of 2× SYBR mix, 1 µL of 1:10 diluted cDNA, 0.4 µM of each primer, and nuclease-free water. The amplification protocol consisted of initial denaturation at 95 °C for 30 s, followed by 40 cycles of 95 °C for 5 s and 60 °C for 30 s. Each biological sample was run in technical triplicate, and amplicon specificity was confirmed via melting curve analysis. Relative transcript abundance was calculated using the 2^−ΔΔCt^ method. Statistical differences among treatments were evaluated using appropriate statistical tests.

## 5. Conclusions

This study provides the first integrated genome-wide framework for the HSP10/Cpn10 co-chaperonin family in *B. juncea*, identifying ten members with distinct genomic organization, predicted subcellular localization, conserved-domain composition, promoter regulatory landscapes, and heat-responsive expression profiles. The predominance of large-scale/segmental duplication events and the non-uniform retention of duplicated loci indicate that polyploid genome evolution has contributed substantially to BjuHSP10 family diversification. Moreover, the abundance of stress- and phytohormone-associated cis-regulatory elements suggests that BjuHSP10 genes may be regulated through multiple environmental and hormonal signaling pathways. Importantly, RT-qPCR analysis provided experimental evidence that BjuHSP10 genes show distinct transcriptional responses to heat stress. In particular, the sustained induction of *BjuHSP10*-4 and *BjuHSP10*-5 and the severe heat-specific activation of *BjuHSP10*-1/3/6/9 identify these genes as priority candidates for investigating chaperonin-mediated thermotolerance in mustard. The RT-qPCR analysis performed in this study provided direct experimental evidence that these BjuHSP10 genes are transcriptionally responsive to heat stress, confirming their heat-responsive expression and providing a functional link between sequence-based predictions and stress-related transcriptional behavior. However, the subcellular localization, structural models, and regulatory mechanisms of these proteins remain largely based on computational predictions and therefore require further functional validation. Future work should use functional assays and gene editing to define their roles in heat tolerance and breeding.

## Figures and Tables

**Figure 1:**
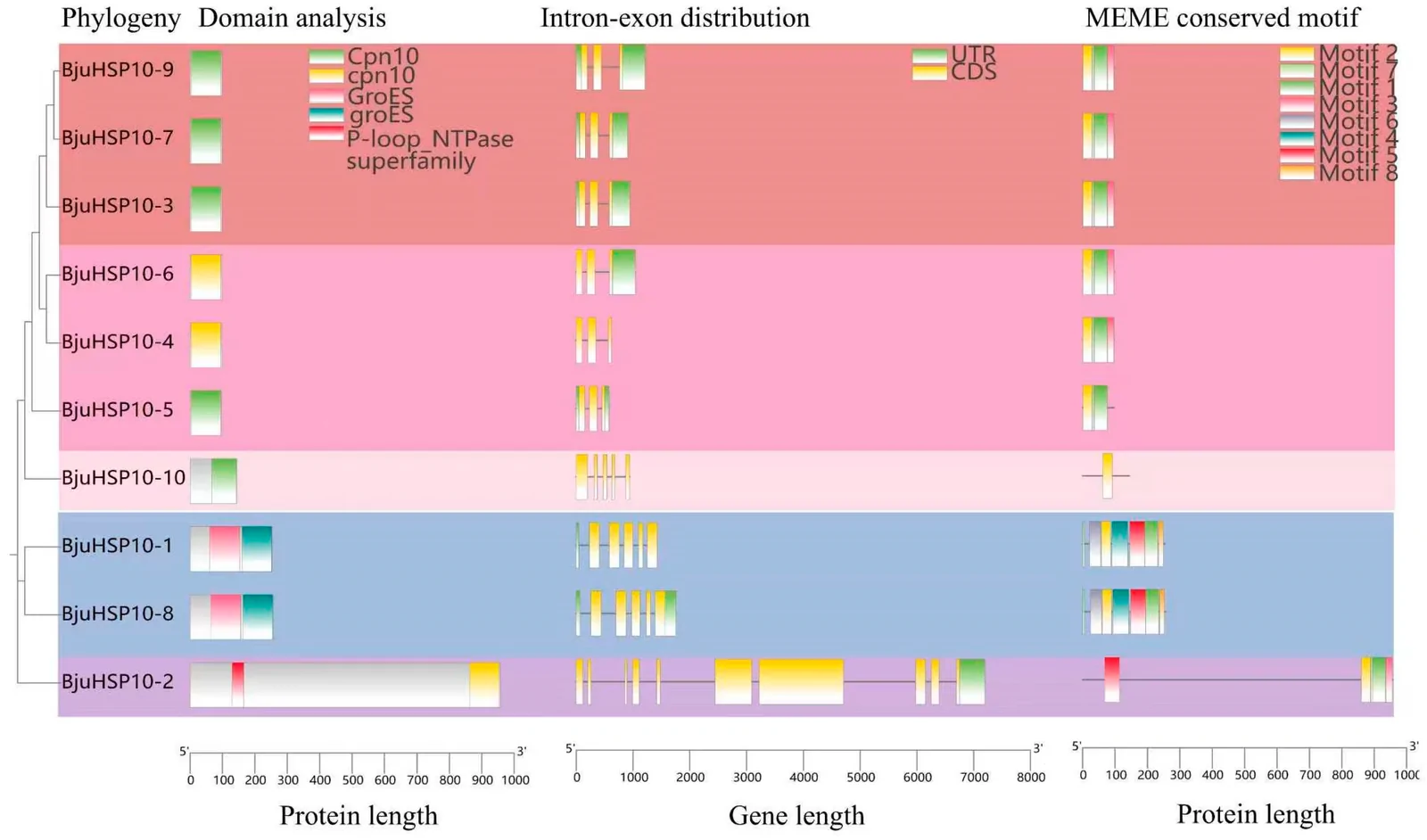
Phylogenetic relationships, conserved domain architecture, gene structure, and conserved motif composition of the *BjuHSP10* gene family. The phylogenetic tree on the left illustrates the evolutionary relationships among the ten identified BjuHSP10 proteins, with distinct shaded backgrounds representing the major phylogenetically clustered groups. The protein domain architecture is shown adjacent to the phylogeny, with conserved CPN10/cpn10, GroES/groES, and P-loop NTPase superfamily domains indicated by different colored blocks according to the domain annotation. The “Cpn10/cpn10” and “GroES/groES” labels represent the same conserved co-chaperonin region identified by two different domain-search models and therefore do not indicate separate functional domains. The middle panel depicts the exon–intron organization of the corresponding *BjuHSP10* genes; CDS and UTR regions are represented by colored and white boxes, respectively, whereas connecting lines represent introns. Gene models are plotted according to their genomic lengths using the scale provided below each panel. The right panel shows the distribution and relative positions of eight conserved motifs (Motifs 1–-8) identified among BjuHSP10 proteins, with each motif represented by a distinct color. The scale bars indicate amino-acid positions for protein features and nucleotide positions for gene structures.

**Figure 2:**
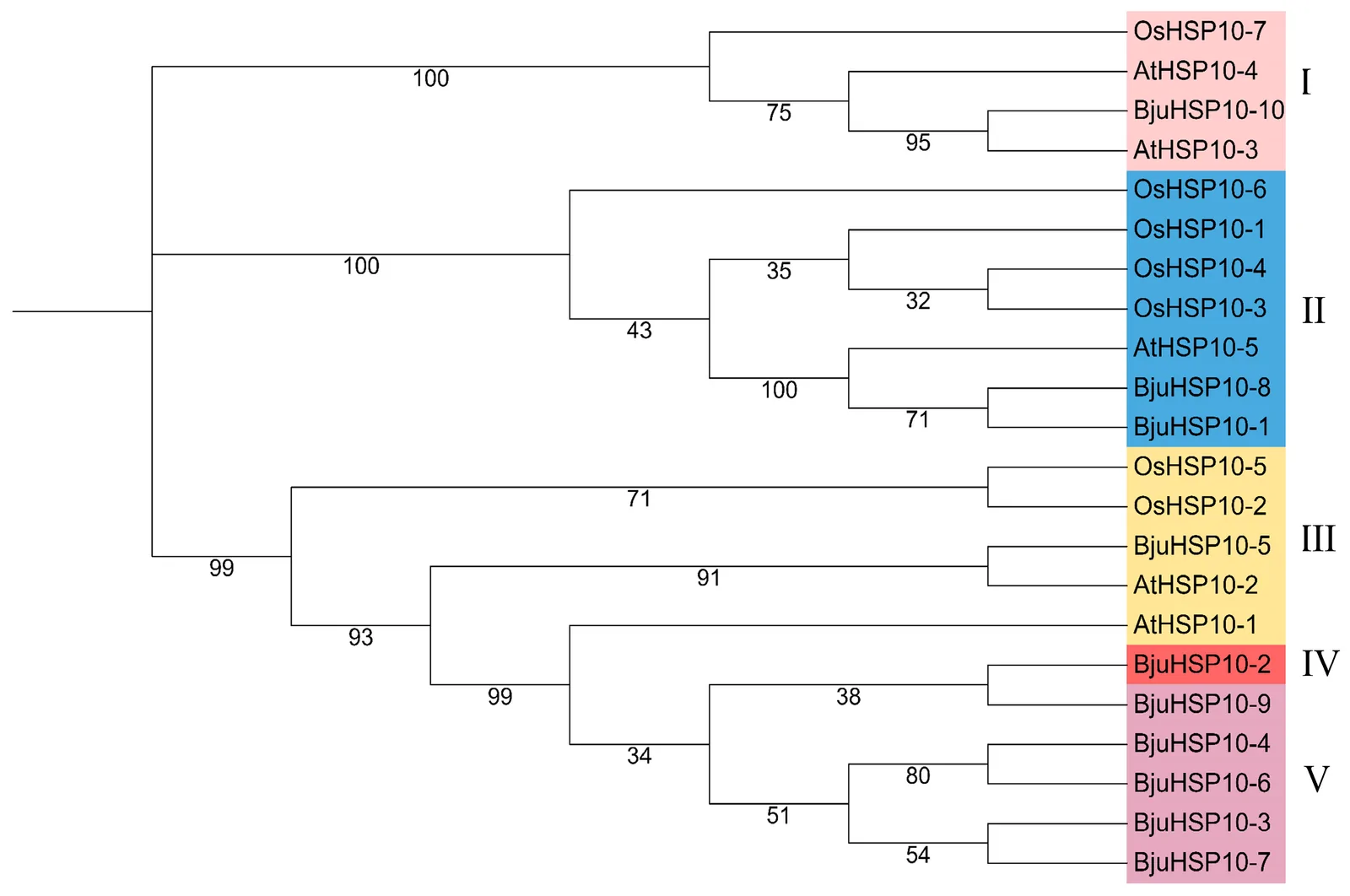
Maximum-likelihood phylogenetic analysis of HSP10-related proteins from *Brassica juncea*, *Arabidopsis thaliana*, and *Oryza sativa*. The phylogenetic tree was reconstructed from a filtered alignment of 96 amino-acid positions representing 22 HSP10-related proteins (ten BjuHSP10, five AtHSP10, and seven OsHSP10) using the maximum-likelihood method under the LG + G + I substitution model. Alignment positions containing gaps in more than 30% of sequences were excluded before tree construction. Bootstrap support values from 1000 replicates are shown at internal nodes. Colored sectors indicate phylogenetic groups. The tree is displayed in a rectangular format.

**Figure 3:**
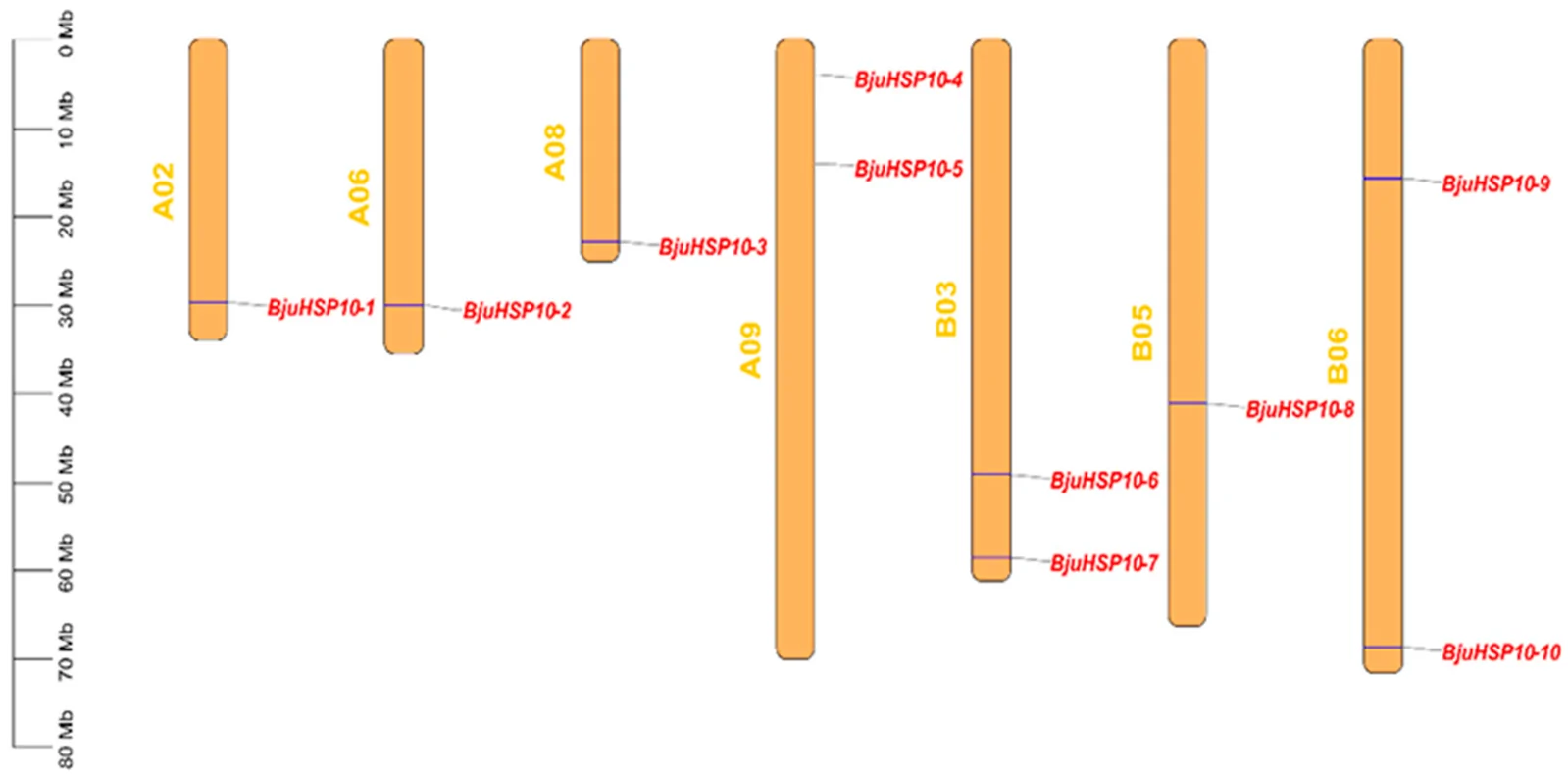
Chromosomal localization and physical distribution of *BjuHSP10* genes across the *B. juncea* genome. The genomic positions of the ten identified *BjuHSP10* genes are shown on the corresponding *B. juncea* chromosomes. Chromosomes are represented by vertical orange bars and are labeled according to the *B. juncea* chromosome nomenclature (A02, A06, A08, A09, B03, B05, and B06). The vertical scale on the left indicates the physical position along each chromosome in megabases (Mb). Horizontal purple lines indicate the approximate genomic positions of individual *BjuHSP10* genes, with gene identifiers shown in red italicized text.

**Figure 4:**
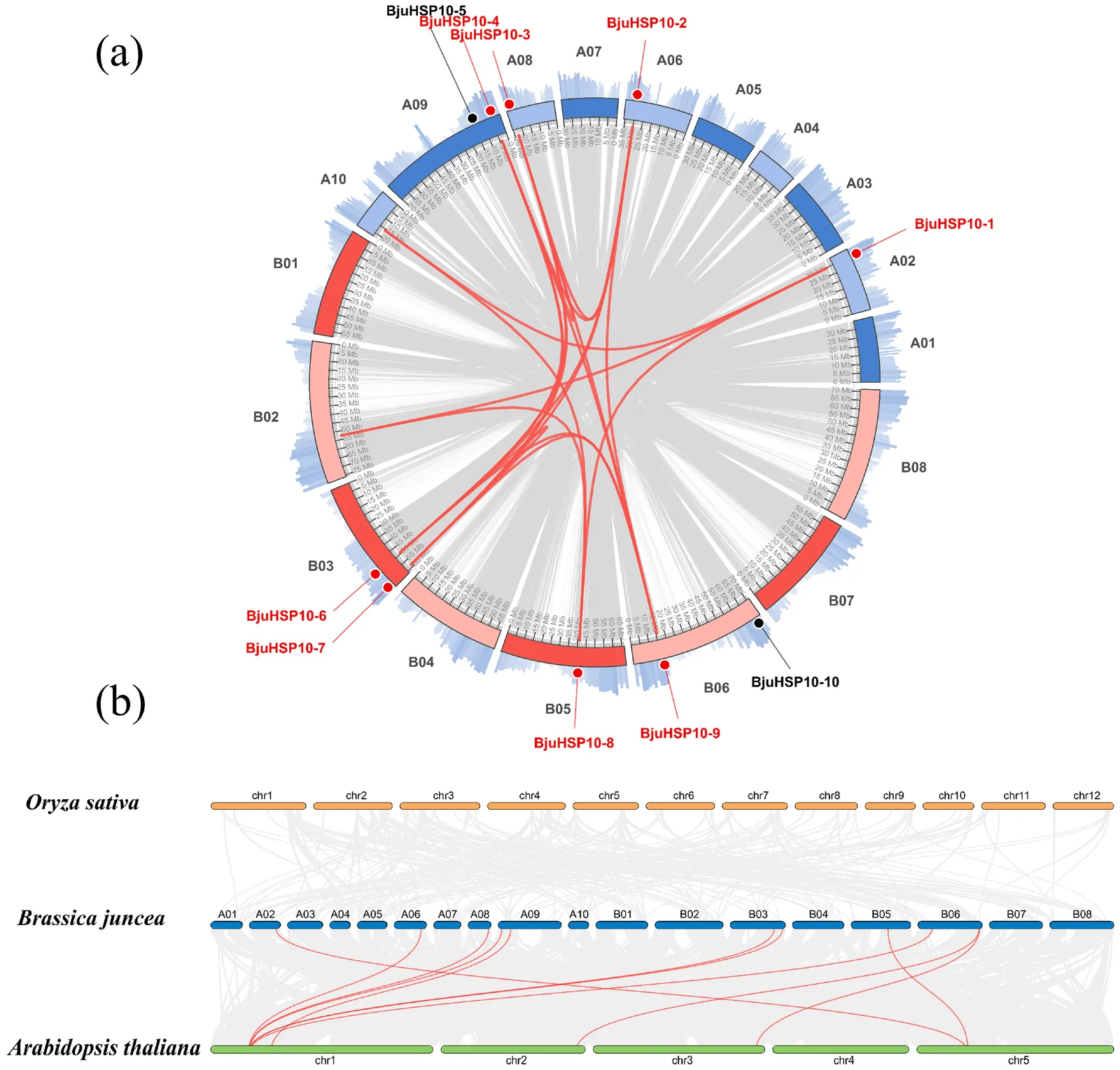
Chromosomal distribution and syntenic relationships of *BjuHSP10* genes. (**a**) A Circos plot illustrating the genomic distribution of the ten *BjuHSP10* genes across the A (A01–A10) and B (B01–B08) subgenomes of *B. juncea*. Chromosomes are arranged according to their physical lengths, with genomic coordinates indicated along the inner chromosome axes. The surrounding blue histograms represent the distribution of genomic features across the chromosomes. *BjuHSP10* loci are marked and labeled according to their chromosomal positions. (**b**) Comparative synteny analysis of *B. juncea* with *O. sativa* and *A. thaliana*. Chromosomes are represented by horizontal-colored bars and labeled according to their respective chromosome nomenclature. Genome-wide collinear relationships are represented by gray ribbons, while red ribbons highlight syntenic relationships associated with *BjuHSP10* loci.

**Figure 5:**
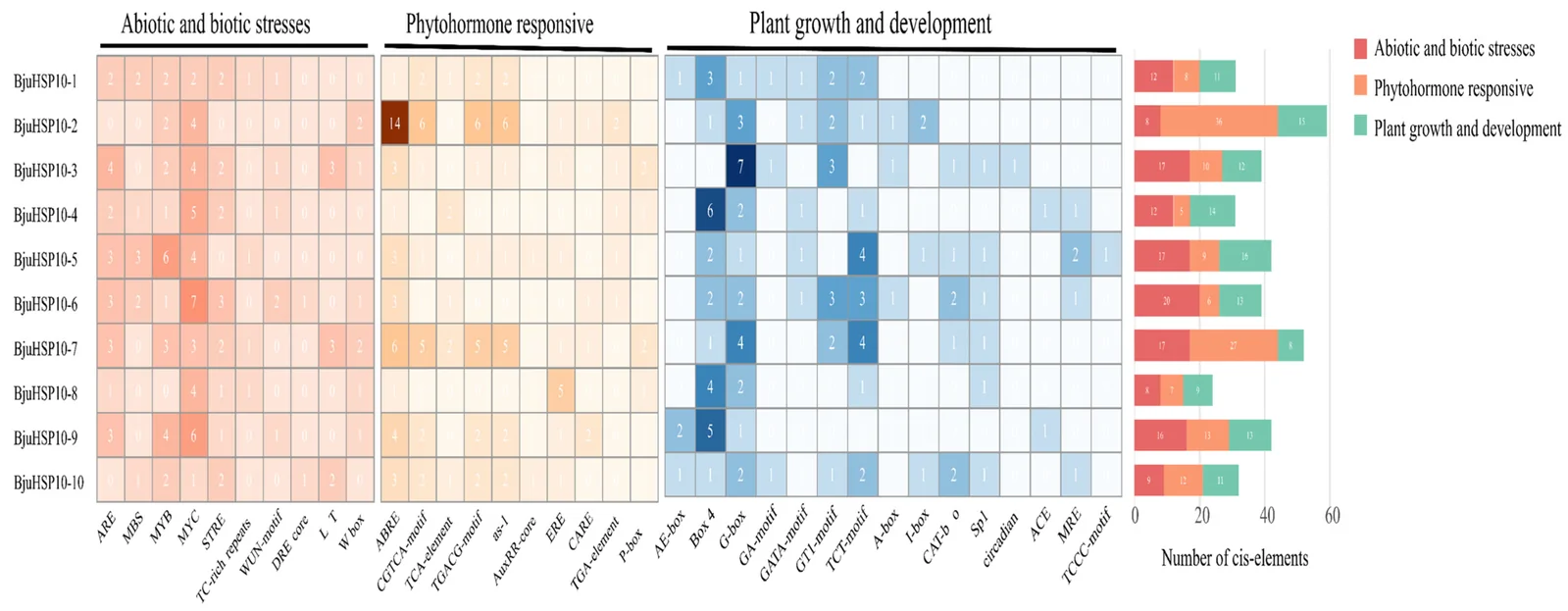
Distribution of cis-regulatory elements in the promoter regions of *B. juncea* HSP10 genes. The heat map shows the occurrence and copy number of predicted cis-regulatory elements in the upstream promoter regions of *BjuHSP10*-1 to *BjuHSP10*-10, grouped into three functional categories: abiotic stress-responsive elements, phytohormone-responsive elements, and plant growth and development-related elements. Each cell represents the number of copies of the indicated motif detected in the corresponding *BjuHSP10* promoter; darker color intensity denotes a higher motif abundance, whereas light or blank cells indicate low or no detected occurrence. The stacked bar chart on the right summarizes the total number of predicted cis-elements in each promoter, with segments colored according to the three functional categories. Numbers within the heat-map cells indicate motif copy number, and the horizontal axis of the bar chart indicates the total number of predicted cis-regulatory elements.

**Figure 6:**
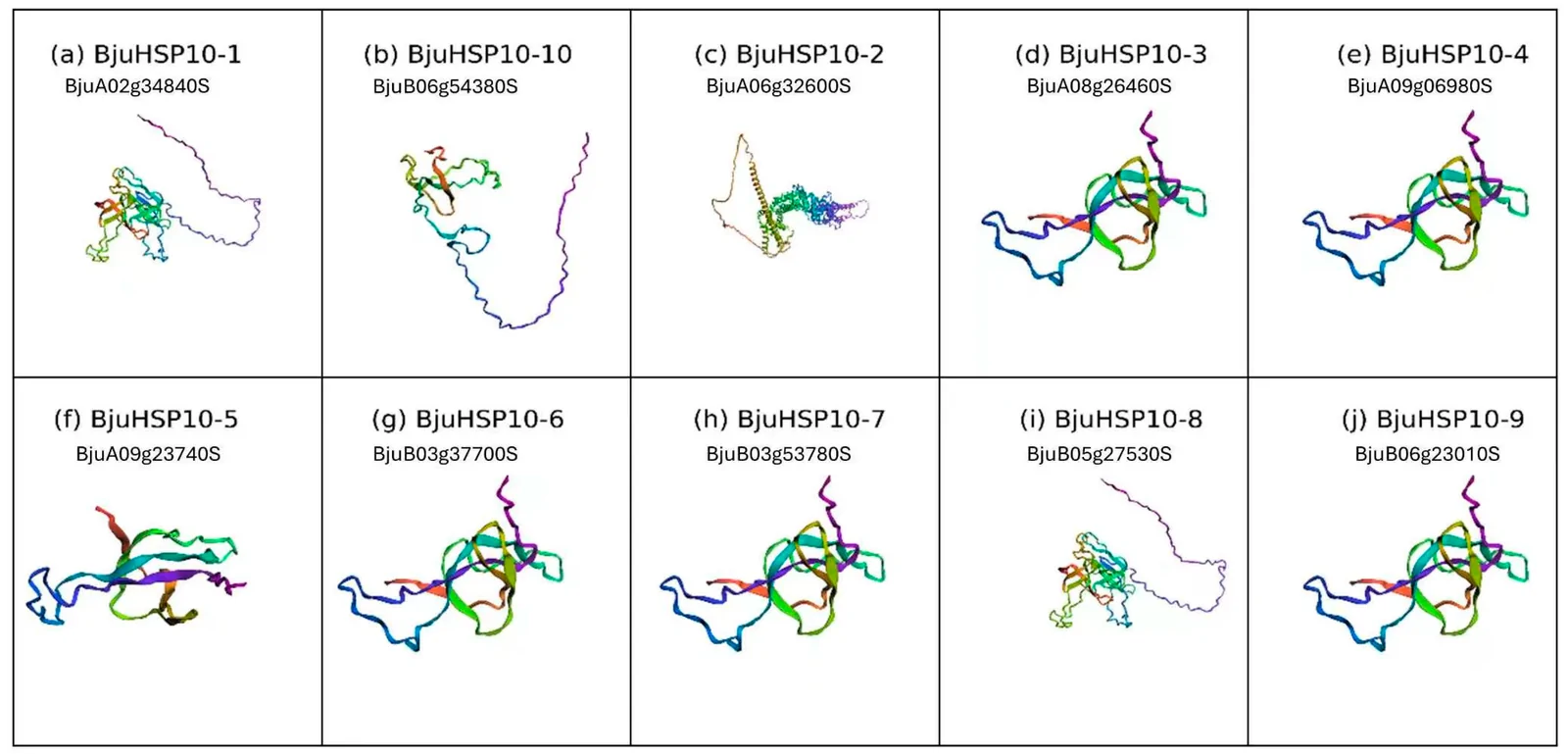
Predicted three-dimensional structures of the *BjuHSP10* protein family in *B. juncea*. Three-dimensional structures of the ten *BjuHSP10* proteins (*BjuHSP10*-1 to *BjuHSP10*-10) were predicted using computational structural modeling and are shown in ribbon representation. The structures are colored in a rainbow gradient according to the amino-acid sequence, with blue representing the N-terminal region and red representing the C-terminal region; intermediate colors indicate the corresponding positions along the polypeptide chain.

**Figure 7:**
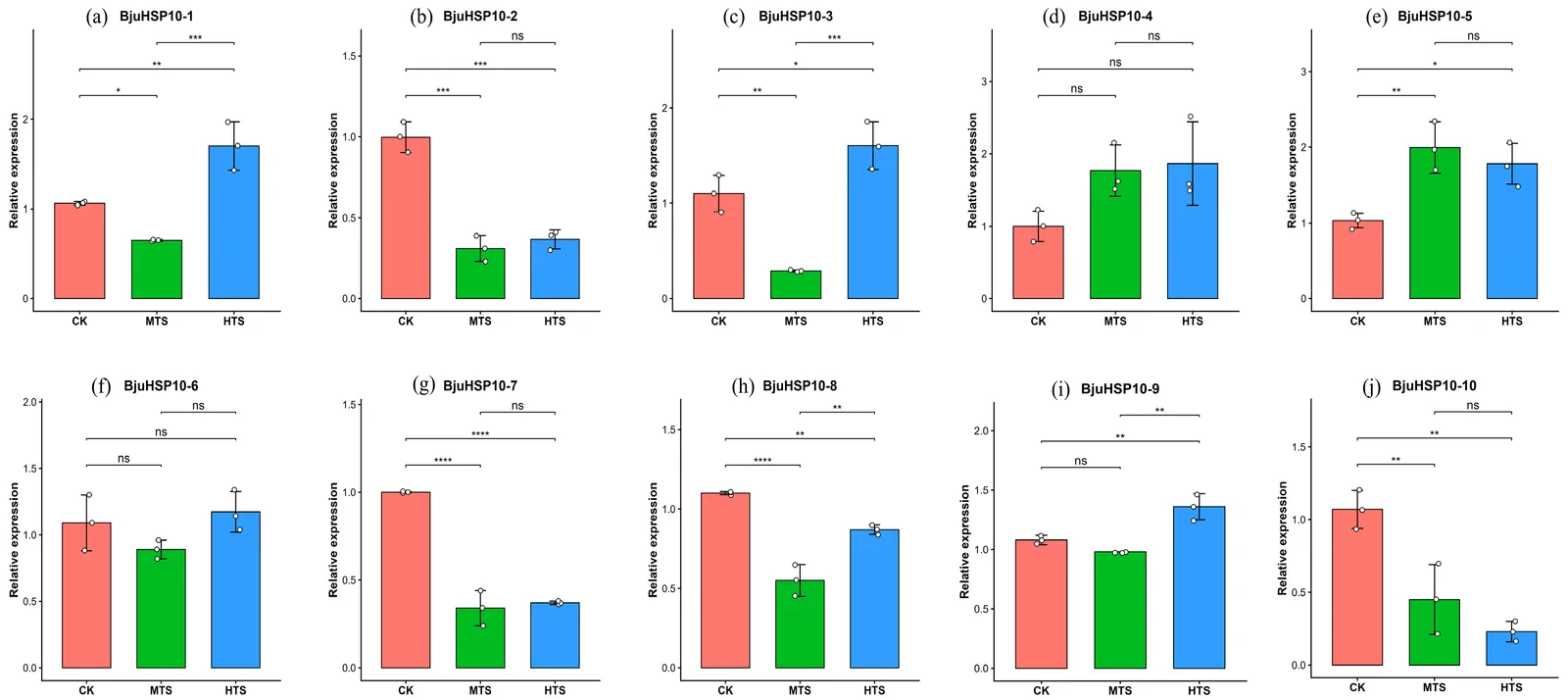
Expression profiles of *BjuHSP10* genes under moderate (MTS) and high temperature stress (HTS). Panels (**a**–**j**) correspond to *BjuHSP10*-1/2/3/4/5/6/7/8/9/10, respectively. Bars represent mean relative expression values ± standard error. Statistical comparisons are indicated by horizontal brackets above the bars. Significance levels are denoted as follows: **** *p* ≤ 0.0001, *** *p* ≤ 0.001, ** *p* ≤ 0.01, * *p* ≤ 0.05, and ‘ns’ for non-significant (Tukey HSD post hoc test).

**Table 1:** Physicochemical characteristics and predicted subcellular localization of *BjuHSP10* genes in *Brassica juncea*.

Gene Name	Sequence ID	Number of Amino Acid	Molecular Weight	Theoretical pI	Instability Index	Aliphatic Index	Grand Average of Hydropathicity	Subcellular Localization
*BjuHSP10*-1	BjuA02g34840S	253	26,797.81	8.87	29.48	97.91	−0.100	Chloroplast
*BjuHSP10*-2	BjuA06g32600S	957	108,783.34	9.35	38.78	94.01	−0.304	Cytoplasm
*BjuHSP10*-3	BjuA08g26460S	98	10,782.50	7.99	30.68	99.29	−0.335	Mitochondria
*BjuHSP10*-4	BjuA09g06980S	98	10,743.38	6.73	39.28	99.29	−0.334	Mitochondria
*BjuHSP10*-5	BjuA09g23740S	97	10,661.32	6.73	35.90	107.42	−0.211	Mitochondria
*BjuHSP10*-6	BjuB03g37700S	98	10,727.38	6.73	37.32	100.31	−0.307	Mitochondria
*BjuHSP10*-7	BjuB03g53780S	98	10,810.56	7.99	30.88	101.33	−0.336	Mitochondria
*BjuHSP10*-8	BjuB05g27530S	256	27,090.14	8.55	28.03	98.24	−0.079	Chloroplast
*BjuHSP10*-9	BjuB06g23010S	98	10,810.56	7.99	28.43	101.33	−0.336	Mitochondria
*BjuHSP10*-10	BjuB06g54380S	144	15,536.74	5.34	39.12	89.31	−0.061	Chloroplast

## Data Availability

The original contributions presented in this study are included in the article/Supplementary Materials. Further inquiries can be directed to the corresponding authors.

## Supplementary Materials

The following supporting information can be downloaded at: https://www.mdpi.com/article/10.3390/ijms27198640/s1.
